# Intensification of short-duration rainfall extremes and implications for flood risk: current state of the art and future directions

**DOI:** 10.1098/rsta.2019.0541

**Published:** 2021-04-19

**Authors:** Hayley J. Fowler, Conrad Wasko, Andreas F. Prein

**Affiliations:** ^1^ School of Engineering, Newcastle University, Newcastle upon Tyne, UK; ^2^ Department of Infrastructure Engineering, The University of Melbourne, Parkville, Australia; ^3^ National Center for Atmospheric Research (NCAR), Boulder, CO, USA

**Keywords:** extreme precipitation, short-duration, flooding, climate change

How to manage the adverse impacts of intensification of rainfall extremes remains an open question. It is known that short-duration (subdaily) rainfall extremes are intensifying as a result of climate change, resulting in increased flood risk, but in order to manage this increased flood risk, the following questions need to be answered:

— By how much are short-duration rainfall extremes intensifying?
— Which processes are dominating rainfall intensification?
— How is flood risk changing as a result of climatological and societal factors? and;
— What is the best way to mitigate and adapt to the changes in short-duration rainfall extremes and the resultant impacts?

INTENSE was the first major international research effort to focus on subdaily rainfall extremes, enabling substantial advances in quantifying historical changes; in combination with a range of modelling efforts, an improved physical understanding of change and hence, a basis for understanding future changes in subdaily rainfall extremes [1]. INTENSE collected a global database of subdaily precipitation data from over 25 000 gauges—the global subdaily rainfall dataset (GSDR) [2] creating the first climatology of hourly rainfall extremes across the globe [3] with a set of freely available indices for subdaily precipitation currently being developed so that this research effort can continue into the future.

Trend analyses in the UK [4] and USA [5] using the GSDR dataset found that trends in winter extremes emerge first in hourly precipitation for both magnitude and frequency statistics and that these trends can be, in part, linked to rising temperatures resulting in a greater moisture-holding capacity in the atmosphere through the thermodynamic Clausius–Clapeyron relationship. Work across the Netherlands has also shown that most hourly precipitation extremes are part of large-scale circulation systems, with considerable forcing from larger scales [6]. These large-scale drivers of hourly precipitation were linked to atmospheric circulation patterns over Europe [7,8], the USA [9], Australia [10] and globally [11], with convection-permitting models (CPMs) over the UK showing that large-scale stability is skilful in predicting the occurrence of subdaily rainfall extremes [12].

CPMs are key to understanding both historical and future subdaily rainfall changes due to their ability to resolve small-scale processes which govern short-duration rainfall intensification [13]. Evaluation of CPMs show the same decrease in the hourly rainfall rates at high surface temperatures seen in observations [12] with subhourly precipitation bearing similar relationships to hourly precipitation [14]. Work on subhourly precipitation extremes from CPMs suggests that storms become more intense over Northern Europe [15] and longer in duration in a warmer climate [14] with more events in autumn months in Europe [16]. CPM simulations over the USA [17] also finds increases in storm size and duration, but results from Australia suggest possible decreases in storm size with higher temperatures [18,19].

Globally, heavy rainfall extremes have been shown to be intensifying with warming at a rate generally consistent with the increase in atmospheric moisture, for accumulation periods from hours to days [20,21]. However, in some regions, high-resolution modelling [22], observed trends [23] and observed temperature dependencies [24] indicate stronger increases in short-duration extreme rainfall intensities than can be expected from atmospheric moisture increases alone. It has been established that at least some of this enhancement of rainfall intensities is from local in-storm effects [22] and urbanization [25]. It is still uncertain what this will mean for future projections of precipitation intensities, due to the unknown effect of large-scale circulation changes [26]. Evidence is emerging that subdaily rainfall intensification is related to an intensification of flash flooding, at least locally [27], but it should be noted that this increase may not be universal due to changes in catchment moisture conditions [28,29].

Extreme heavy precipitation is increasing in frequency and intensity, and results from new global subdaily datasets and high-resolution modelling suggest that subdaily precipitation extremes might intensify more than anticipated based upon thermodynamic considerations. Hence, understanding the processes driving change in various climate zones and their variations on regional scales is vital in advancing the projection of rainfall extremes. The INTENSE project culminated with a discussion meeting on the intensification of short-duration rainfall extremes and implications for flash flood risks with a focus on process understanding and integrating/synthesizing research results from different international groups working in this area. The articles in this special issue present research from a diverse group of climate scientists, meteorologists, hydrologists, atmospheric physicists, engineers and practitioners from across the world focusing on understanding changes in short-duration rainfall extremes, and how their potential impacts can be managed through practical flood guidance.

The special issue opens with a summary of the current scientific knowledge of climate change impacts on short-duration rainfall extremes led by Fowler and co-authored by the meeting participants [30]. This article summarizes the current understanding of short-duration rainfall extremes from the perspectives of high-resolution climate models and empirical evidence using observations. It concludes that this foundational knowledge must continue to be developed through international collaboration if the mechanisms of rainfall change are to be fully understood and enable meaningful attribution studies to be performed. The issue then presents an attribution study by Villarini & Zhang [31], which shows that increases in spring precipitation across the Central United States are caused by rising greenhouse gases. This article forms part of the growing, but still emerging, body of literature linking anthropogenic climate change directly to changes in rainfall. Linking weather patterns to rainfall events is important for understanding and unravelling the thermodynamic (increase in temperature and moisture) and dynamic (changes in large-scale circulation) contributions to extreme weather events. Moron *et al*. [32] perform weather type classification and trend analysis across boreal monsoonal India to reveal the contributions of each weather type and enhance our understanding of their relative importance to changes in subdaily rainfall extremes.

CPMs form a crucial line of evidence by increasing our understanding of the processes governing changes in short-duration rainfall extremes and enabling improved climate change projections. Here, Wehner *et al*. [33] provide confidence in the accuracy of a novel set of high-resolution global models (HighresMIP) [34] by evaluating simulated subdaily extreme rainfalls against observed data. O'Gorman *et al*. [35] present state-of-the-art modelling experiments in response to uniform warming to show an intensification of short-duration rainfall extremes. Kendon *et al*. [36] then summarizes the challenges and outlook for convection-permitting climate modelling, suggesting a way forward where improvements can be made reducing the uncertainty in our future projections of subdaily rainfall extremes.

The empirical relationship between extreme rainfall and observed temperature forms an important line of evidence for changes in short-duration rainfall extremes. This relationship is further examined by Lenderink *et al*. [37] in a set of climate experiments using a CPM, finding that current climate scaling relations between rainfall and temperature reasonably predict future relations. Prein *et al*. [38] show that CPMs can reliably capture the large-scale features of organized convective storms and corresponding climate change signals, including extreme precipitation changes compared to large-eddy simulations (250 m grid spacing). The application of such high-resolution modelling efforts in water management is then examined by Orr *et al*. [39] and, as many idealized experiments use temperature forcing to represent anthropogenic climate change, Wasko [40] reviews the application of temperature sensitivities for informing changes to flood extremes with global warming.

To understand how best to adapt flood management practice for climate change, Wasko *et al*. [41] review current practice around the world, finding that although globally efforts are emerging to manage flood impacts of climate change, there remains a focus on extreme rainfall changes with little consideration given to other factors such as antecedent moisture conditions. Based on the evidence of a two-stepped change in rainfall extremes (both thermodynamic and dynamic), Sharma *et al*. [42] then put forward a possible strategy for engineering design and water management in a changing world. Finally, Dale [43] presents a pragmatic practitioner's view of what can be done to manage the effects of increasing extreme subdaily rainfall and their impact of raising the risk of flash flooding.

As evidence of increases in short-duration rainfall extremes continues to emerge, above those that could be expected from thermodynamic increases in moisture alone, there remains a need to both quantify the increase and understand the relative thermodynamic and dynamic contributions. With the strong link to atmospheric circulations acting as a precursor for short-duration rainfall extremes in subtropical regions, the changing nature of those systemics driving short-duration rainfalls must also be understood and quantified. Observational evidence and CPM model experiments suggest convective cloud feedbacks can cause rainfall intensification above what could be expected by thermodynamics alone, but the nature of this increase is still uncertain. There remains a need for continued investigations and improved understanding of mechanisms that cause changes in subdaily extreme rainfalls, which will only be possible by dedicated observational efforts merged with further development of CPMs.

With the intensification of subdaily extreme rainfall expected to increase flood risk, in particular flash flooding in urban areas, there exists a substantial challenge in developing adaptation strategies. Flood guidance is moving towards adaptive decision-making, but examples of this remain isolated, with little cohesion across jurisdictions on how to manage changes in the inputs to water management decisions. This points to the need for renewed global efforts, such as INTENSE, which bring together scientists, practitioners and policy-makers from around the world to ensure that our understanding of changes in subdaily rainfall extremes continues to improve and required changes to water management are readily implemented.

## Data accessibility

This article has no additional data.
